# Cellulose Based Photonic Materials Displaying Direction Modulated Photoluminescence

**DOI:** 10.3389/fbioe.2021.617328

**Published:** 2021-03-30

**Authors:** Molíria V. Santos, Fernando E. Maturi, Édison Pecoraro, Hernane S. Barud, Laís R. Lima, Rute A. S. Ferreira, Luís D. Carlos, Sidney J. L. Ribeiro

**Affiliations:** ^1^Institute of Chemistry, São Paulo State University (UNESP), Araraquara, Brazil; ^2^Department of Physics, CICECO – Aveiro Institute of Materials, University of Aveiro, Aveiro, Portugal; ^3^Biopolymers and Biomaterials Laboratory, University of Araraquara, Araraquara, Brazil

**Keywords:** bacterial cellulose nanocrystals, chiral nematic liquid crystal, iridescence, photonic materials, luminescence, modulated light emission, rhodamine 6G

## Abstract

Photonic materials featuring simultaneous iridescence and light emission are an attractive alternative for designing novel optical devices. The luminescence study of a new optical material that integrates light emission and iridescence through liquid crystal self-assembly of cellulose nanocrystal-template silica approach is herein presented. These materials containing Rhodamine 6G were obtained as freestanding composite films with a chiral nematic organization. The scanning electron microscopy confirms that the cellulose nanocrystal film structure comprises multi-domain Bragg reflectors and the optical properties of these films can be tuned through changes in the relative content of silica/cellulose nanocrystals. Moreover, the incorporation of the light-emitting compound allows a complementary control of the optical properties. Overall, such findings demonstrated that the photonic structure plays the role of direction-dependent inner-filter, causing selective suppression of the light emitted with angle-dependent detection.

## Introduction

In the past years, the use of photonic crystals for manipulating light has taken the development of optical devices to a whole new level (Abdollahramezani et al., 2020). The characteristic structural colors seen in such materials originate from the interaction of light with their periodic structure, such as diffraction, interference, scattering, or polarization (Liu et al., 2020). The structure-dependent color is more advantageous when compared to colors produced by pigments or dyes because it can be tuned by controlling the crystal morphology and it will not vanish over time (Boott et al., 2020; Xu et al., 2021). Despite most photonic crystals used in optoelectronics are obtained from synthetic materials, biomaterials are responsible for producing structural colors in different animals and plants (Tran et al., 2020).

Cellulose stands out among the myriad of biomaterials available because it is a renewable biopolymer and it is the most abundant raw material for the production of photonic crystals (Dumanli et al., 2014). The acid hydrolysis can be performed on different sources of cellulose [e.g., plants (Beck-Candanedo et al., 2005; Habibi et al., 2008; Siqueira et al., 2009), bacteria (Roman and Winter, 2004), and tunicates (Favier et al., 1995)] by using sulfuric acid to obtain stable aqueous suspensions of needle-like cellulose nanocrystals (CNC) that selectively reflect polarized light (Eichhorn, 2011; Klemm et al., 2011). Above a certain critical concentration, CNC dispersed in water self-assembly into a lyotropic cholesteric liquid crystalline phase that can be preserved upon slow drying, resulting in iridescent films (Revol et al., 1992, 1998; Arrighi et al., 2002). The iridescence occurs because the CNC layers are stacked in a long-range assembly forming a helicoidal pitch (*P*) with a characteristic 360° degree rotation within the same length scale of the visible light (de Vries, 1951; Habibi et al., 2010; Mendoza-Galván et al., 2018), paving the way for the development of tunable filters (Driesen et al., 2007; Penninck et al., 2012), polarizing mirrors (Broer et al., 1995), reflective displays (Yang et al., 1994), and lasers (Kopp et al., 1998). However, CNC films are brittle materials and present poor mechanical properties, which is the main drawback of using pristine CNC (Niinivaara and Cranston, 2020).

Nevertheless, the self-assembly of CNC is still interesting because it can be used to produce freestanding inorganic films with chiral nematic structure by incorporating inorganic precursors into CNC suspensions, controlling the size and organization of template-driven structures, such as silica photonic crystals and organosilica films (Shopsowitz et al., 2012; Kelly et al., 2013, 2014; Habibi, 2014; Van Opdenbosch and Zollfrank, 2014; Hiratani et al., 2017). After the removal of CNC, mesoporous solid structures are achieved with high surface area and long helical twist range. Both helical pitch and porosity can be tailored by changing the relative content of silica precursors, tuning the chiral nematic structure (Shopsowitz et al., 2010; Shopsowitz et al., 2014). Furthermore, the modulation of light emission can be assessed by incorporating light-emitting compounds within the ordered structure of CNC, allowing applications such as anti-counterfeiting in documents, optical memories, and biochemical sensing (Chu et al., 2014a, b, 2015; Nguyen et al., 2014; Qu et al., 2016; Nguyen et al., 2017; Lin et al., 2018).

Recently, some of us have demonstrated a novel strategy to modulate the narrowband emission of trivalent lanthanide ions complexes by using CNC films for applications in sensors, lasers, and tunable filters (Santos et al., 2019). Herein, we report the preparation of luminescent iridescent films obtained by combining tetraethoxysilane (TEOS), a CNC suspension obtained from bacterial cellulose, and a light-emitting organic dye (Rhodamine 6G, Rh6G). The influence of the angle-dependent reflection of light on the emission properties was systematically studied and we evaluated the benefit of the simultaneous integration of light emission and photonic structure of the obtained CNC-based composite films.

## Materials and Methods

### Production of Bacterial Cellulose Membranes

Bacterial cellulose (BC) was prepared according to a previously reported procedure (Barud et al., 2011; Tercjak et al., 2015). BC membranes were obtained from an isolated culture of *Gluconacetobacter xylinus* (American Type Culture Collection, ATCC 23769, 5 mL). The sterile culture medium (glucose, 50 g L^–1^; yeast extracts, 4 g L^–1^; anhydrous disodium phosphate, 2 g L^–1^; heptahydrate magnesium sulfate, 0.8 g L^–1^; and ethanol, 20 g L^–1^) was kept at 28°C in plastic trays (30 × 50 cm^2^). After 96 h, the obtained BC membranes (3 mm thick, 1 wt% cellulose and 99 wt% water) were washed several times with deionized water. An aqueous solution of NaOH (1 wt%, 70°C) was added until pH = 7.0 to remove the remaining bacteria. Dried BC membranes were obtained after drying at 80°C over Teflon^®^ supports.

### Preparation of Cellulose Nanocrystals

The preparation of CNC followed a previously reported method (Mendoza-Galván et al., 2018). Previously dried BC membranes (5 g) were milled using an IKA^®^ A11 basic analytical mill and sieved with a stainless-steel sieve (mesh 35). The milled BC was hydrolyzed in sulfuric acid (Synth 98%, 88 mL, 64 wt% concentration) at 50°C for 0.5 h under vigorous stirring. The obtained suspension was diluted (1:10) in ultrapure cold water to stop the hydrolysis, and it was allowed to settle overnight. The clear top layer was decanted and the remaining cloudy layer was centrifuged at 6,000 rpm for 10 min (Jouan C3i—CR3i multifunction centrifuge). The supernatant was decanted and the resulting thick white suspension was washed three times with ultrapure water. The obtained suspension was dialyzed against ultrapure water (cellulose dialysis tubing, 12,000–14,000 MWCO) until constant pH = 2.4. The suspension was diluted to 3.0 wt% and dispersed using an ultrasound homogenizer (Sonics Vibra-Cell VC 505 500W 20 kHz) with a 6 mm diameter probe. The CNC suspension (3.0 wt%, 50 mL) was transferred to a 100 mL plastic tube and sonicated at 60% of the maximum power (300 W). Additionally, a prolonged sonication (energy input of 7,500 J g^–1^ of CNC) was performed in an ice bath to prevent desulfation caused by the heating generated during the process.

### Preparation of Nanocrystalline CNC-Silica Composite Films

Different amounts (33 μL, 66 μL, 98 μL, 131 μL) of TEOS (Sigma-Aldrich 98%) were added to the obtained aqueous suspension of CNC (3.0 wt%, 4 mL) and the resulting mixtures were stirred at room temperature for 3 h, leading to homogeneous mixtures. Freestanding CNC/silica composites films were obtained by drying the solutions onto polystyrene Petri dishes (Supplementary Figure S1). Films were named according to the relative content of CNC/silica: 0% (CNC), 12.5 wt% (CS1), 25.0 wt% (CS2), 33.3 wt% (CS3), and 50.0 wt% (CS4).

### Preparation of Nanocrystalline Photoluminescent CNC-Silica Composite Films

An ethanolic solution of Rh6G (200 μL, 10^–4^ mol L^–1^) was added to the homogeneous mixture containing CNC and TEOS. The obtained mixtures were transferred to polystyrene Petri dishes, allowing its evaporation at room temperature until freestanding films were formed. The obtained composite films can be seen in Supplementary Figure S2.

### Characterization of CNC and Composite Films

Thermogravimetric analysis (TGA) was performed using a Shimadzu TGA-50 system, in the temperature range from 25 to 800°C at a heating rate of 10°C min^–1^, under a static atmosphere of air. Polarized optical microscopy (POM) images were obtained with an Olympus BX41 microscope, using crossed polarizers. Scanning Electronic Microscopy (SEM) images were registered with a Hitachi SU-70 electron microscope. The samples were attached to aluminum stubs using double-sided carbon adhesive tape or carbon glue. Pictures were obtained from the surface and cross-section areas. All the samples were sputter-coated with carbon through an EMITECH K950X Turbo Evaporator, at a single pulse, on an outgassing time of 30 s and an evaporating time of 2 s. Transmission electron microscopy (TEM) images were obtained by a Philips CM200 microscope with an accelerating potential of 100 keV. TEM samples were prepared using a method described by Giasson et al. (1988).

UV-Vis reflectance spectroscopy was carried out using a Perkin-Elmer Lambda 950 UV/Vis/NIR spectrophotometer and a Spectralon integrating sphere (Ø = 150 mm). The freestanding film surface was placed perpendicularly to the incident beam and the spectra were acquired as a function of the incident angle (15° < θ < 60°) with 5° steps, as illustrated in Supplementary Scheme 1.

### Photoluminescence

Excitation and emission spectra were recorded at room temperature with a modular double grating excitation spectrofluorometer through a TRIAX 320 emission monochromator (Fluorolog-3, Horiba Scientific), coupled to an R928 Hamamatsu photomultiplier using the front face acquisition mode with a 450 W Xe arc lamp.

The angle-dependent emission spectra were recorded at room temperature as a function of the detection angle (0° < θ < –90° and 0° < θ < 90°) through a modular experimental setup, illustrated in Supplementary Scheme 2. The modular experimental setup consists of an Ocean Optics HR2000+ES USB spectrometer and a jacketed optical fiber (Ocean Optics) QP600-UV-Vis (core diameter of 600 mm and a total length of 1 m) to collect the emission signal. A diode laser (405 nm, CW mode, 5 mW, FWHM = 4 nm) was used as the excitation source. The laser beam was directed at an angle of 45° regarding the detection plane, and the optical fiber tip was aligned perpendicularly to the surface of the sample. At last, the sample holder was fixed at the center of the rotatory base with a goniometer and the tip of the fiber collected the emitted signal, allowing free rotation around it. The emission quantum yield (Φ) was measured at room temperature in a Quantaurus-QY Plus C13534 (Hamamatsu) system with a 150 W xenon lamp coupled to a monochromator, an integrating sphere, and two multichannel analyzers to record the emission intensity. For each sample, the reported Φ value is he average values of three measurements with an accuracy of 10%.

## Results and Discussion

**FIGURE 1:**
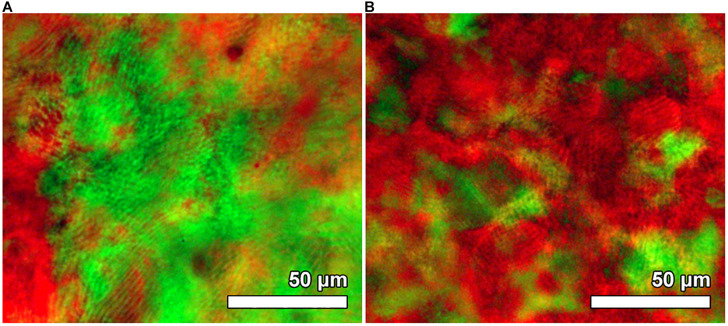
POM image of sample CS1-Rh **(A)** and CS4-Rh **(B)** between crossed polarizers.

The Rh6G doped CNC/silica composite films were obtained with 0, 8.5, 15.4, 20.7, and 35.2 wt% of silica (from TGA, Supplementary Figure S3) and named as CNC-Rh, CS1-Rh, CS2-Rh, CS3-Rh, and CS4-Rh, respectively. After a controlled drying, the chiral nematic phase was retained in the composites films, which display strong iridescence under non-polarized room light exposure (Supplementary Figure S2). POM images (Figure 1) of the composite films show a redshift of the color with increasing relative silica content while the overall texture remained essentially unchanged. The characteristic fingerprint lines of chiral nematic structures were observed orthogonally to the chiral nematic axis, with the shortest distance between lines equals half a pitch (*P*/2) of the cholesteric helix (Arrighi et al., 2002). The *P*/2 values obtained from Figure 1 range from 2 to 3 μm, which are in good agreement with values reported for CNC films (Majoinen et al., 2012; Lagerwall et al., 2014).

**FIGURE 2:**
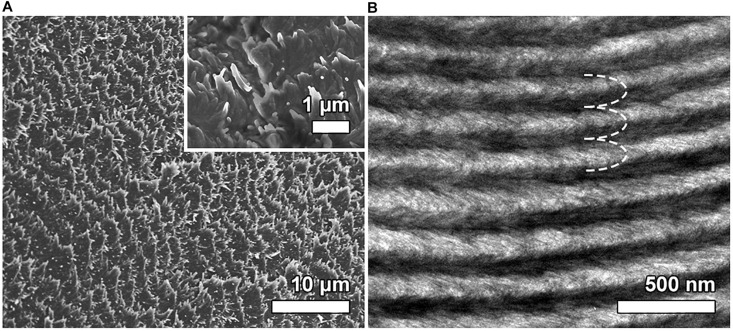
SEM image of the surface view of the CS4-Rh sample **(A)**. The inset panel in A displays an SEM image with higher magnification highlighting the left-handed rotation of the chiral nematic organization. TEM image of an oblique cross-section of the CS4-Rh composite **(B)**. The dashed white line shows the arc-like appearance generated through the chiral nematic structure.

Electron micrographs in Figure 2 provided further confirmation of the preservation of chiral nematic organization after the incorporation of silica and Rh6G into the CNC structure. The top view of the surface of the CS4-Rh composite film (Figure 2A) shows a multilayered fan-like structure rotated in the counter-clockwise direction, indicating that nanocrystals present left-handed helicoids, as expected for chiral nematic helicoidal arrangements in a CNC-based structure (Wilts et al., 2017). The TEM image of a fractured cross-section of the film (Figure 2B) shows that the periodic structure is seen throughout the entire thickness of the film, in which each repeating layer gives rise to the arcing effect due to the 180° rotation of the chiral nematic direction (Arrighi et al., 2002; Majoinen et al., 2012; Zhanga et al., 2013).

**FIGURE 3:**
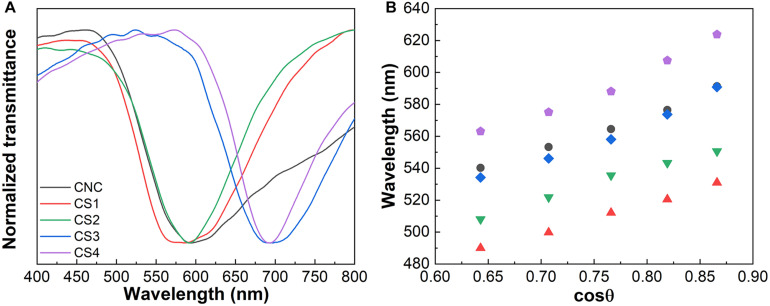
Transmittance spectra of CNC, CS1, CS2, CS3, and CS4 samples **(A)**. Variation of the peak position of the reflectance spectra as a function of the cosθ for the same CNC/silica composites **(B)**.

**FIGURE 4:**
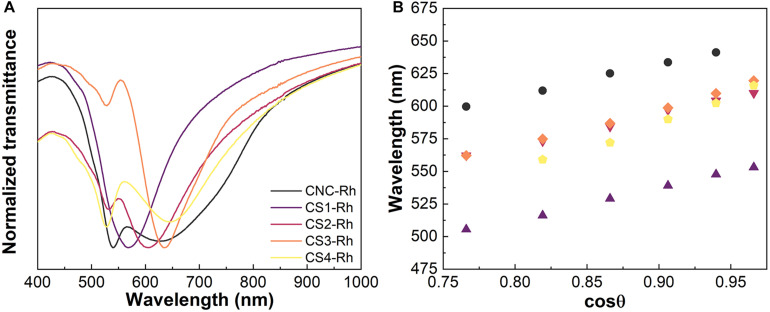
Transmittance spectra of CNC, CS1-Rh, CS2-Rh, CS3-Rh, and CS4-Rh **(A)**. Variation of the peak position of the reflectance spectra as a function of the cosθ for the same CNC/silica composites doped with Rh6G **(B)**.

Transmittance spectra of CNC/silica composite films (Figure 3) were obtained at a normal incident angle (θ = 0°), where the Bragg diffraction peaks are seen in the visible part of the spectra between 550 and 700 nm. The transmittance spectra obtained for the Rh6G containing CNC/silica composites (Figure 4) present an additional absorption band peaking around 530 nm, which refers to the absorption of Rh6G (Martnez et al., 2005). These results are consistent with the formation of fluorescent J-type dimers due to the aggregation of Rh6G molecules when encapsulated into solid composite films (Martínez Martínez et al., 2005; Anedda et al., 2007), where the absorption band of the dye is shifted to lower energies by increasing the concentration of Rh6G (Lofaj et al., 2013).

Reflectance spectra were recorded as a function of the incident angle (20° < θ < 60°, Supplementary Figure S4). Figures 3B, 4B display the variation of the peak position in the reflectance spectra as a function of the cosθ. The spectra show that Bragg diffraction is taking place, presenting a characteristic maximum wavelength (λ_0_) which obeys the Vries’ equation Eq. 1 (de Vries, 1951; Eichhorn, 2011):

(1) $$\lambda_{0}={\text{n}}_{ave}\text{P}cos\theta$$

where θ is the incident angle between the normal surface of the sample and the incident ray, and *n*_*ave*_ is the average refractive index. In this case, the peaks observed in the reflectance spectra shift to shorter wavelengths as the viewing angle increases relatively to normal on the film. The specular reflectance spectra of the CS4-Rh composite (Supplementary Figure S4B) show a peak centered at 528 nm for θ > 50° due to the re-absorption process, i.e., part of the reflected light is absorbed by the dye molecules. Similar behavior was observed for all the samples containing Rh6G.

It is possible to calculate *l*_0_ through Vries’ equation by using the values of *P* obtained from the chiral nematic structure. These values were determined from SEM images of cross-sectional views of the obtained composite films (Supplementary Table S1). The values of *P* depend on the relative amount of silica and at least two groups with similar values of *P* may be identified for lower and higher silica content. The pristine CNC presents a larger value of *P* than the values observed for samples with lower silica content, indicating that changes in *P* are caused by the formation of thicker nanocrystal walls after the incorporation of silica resulting from ionic interactions between negatively charged silica species and crystalline cellulose. This is supported by the band narrowing in the transmittance spectra of CNC/silica composites after the addition of silica (Figure 4A), which demonstrates an improvement of the chiral nematic organization and a better definition of *P* by increasing the silica content. The same behavior was observed for composites containing Rh6G, although the presence of Rh6G contributed to further increasing the values of *P* when compared to the composites without dye. Therefore, a complementary control of the optical properties is seen.

According to the analysis performed on the results presented in Figure 2B, there is a regular spacing corresponding to the *P*/2 value of 115 ± 3 nm for the CS4-Rh composite. This value is comparable to the one obtained for *P*, which can be calculated using the Vries’ equation (*P* = 240 ± 12 nm). However, the texture is rich in parallel lines, with a distance of about 2–3 mm, suggesting that *P* is one order of magnitude larger than the Bragg reflection of visible light. Such intriguing superposed phenomena and apparent contradictions were discussed recently by Lagerwall et al. (2014). They proposed an explanation for the observation discussed above, taking into account the possible non-uniform drying at the surface and the core. During the drying process, the CNC concentration increases rapidly at the surface as the water evaporates leading to the helix development on the film plane, with *P* in the micrometer range. Nevertheless, in the core, the sample is still in a liquid crystalline state and the diffusion decreases drastically because the surface regime has been solidified. Hence, the increase in the core concentration is much slower than at the surface. In the core, the CNC concentration could increase further, yielding a sufficiently short pitch to produce a photonic bandgap in the visible wavelength range, as it is observed in the composite films.

**FIGURE 5:**
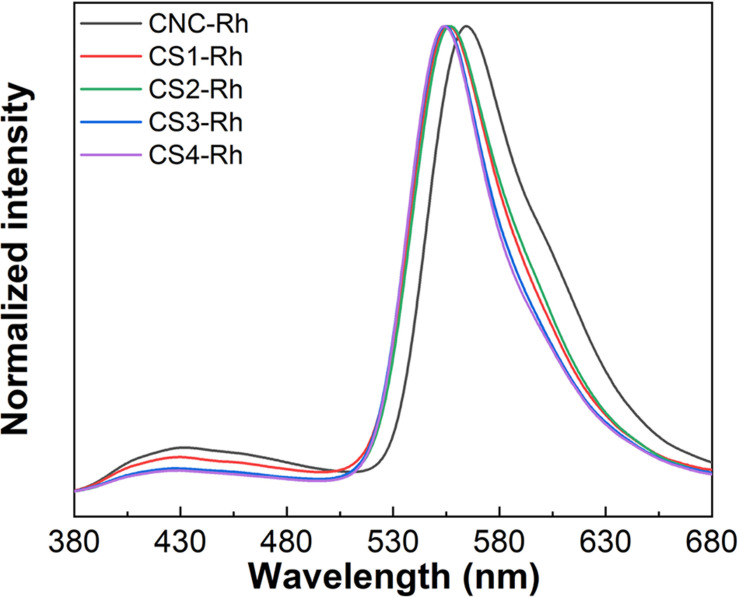
Emission spectra of CNC/silica composite films doped with Rh6G under excitation at 348 nm.

The emission spectra of Rh6G-doped CNC/silica composites presented in Figure 5 show that two emission bands can be seen under excitation at 345 nm. The first one corresponds to the emission of cellulose nanocrystals peaking around 430 nm (Li et al., 2009), confirmed by the emission spectrum of pristine CNC under excited at 348 nm (Supplementary Figure S5). The second one displays the characteristic emission of Rh6G, which is dependent on the relative dye content (Anedda et al., 2005). At lower concentrations, monomeric properties of Rh6G are observed, with an emission band centered around 550 nm (Bujdák, 2006; Carbonaro et al., 2009). The emission spectra in Figure 5 display a band centered around 556 nm and a shoulder above 605 nm. Besides that, the emission spectrum of the sample without silica (CNC-Rh) showed a band centered at 565 nm and a shoulder above 610 nm. Bathochromic spectral shift related to the monomer can be attributed to the formation of fluorescent dimers (J-type). Usually, at relatively high concentrations of Rh6G, fluorescent (J-type) and non-fluorescent (H-type) dimers are formed (Bujdák, 2006). Additionally, the dye aggregation leads to a concentration quenching of the luminescence of Rh6G (Martnez et al., 2005). The emission of J-type dimers is red-shifted in comparison to the monomer emission and the total emission spectra showed an overlap with emission spectra from the two molecular species. These results can also be attributed to changes in the chemical environment in their surroundings and the decrease in loading efficiency of Rh6G while the content of silica increases, which was observed in the transmittance spectra.

The emission lifetime and quantum yield values for CNC, CNC-Rh, and CS-Rh samples are shown in Table 1. The emission lifetime value of pristine CNC film is 6.83 ± 0.05 ns, which increases by increasing the relative content of silica due to the overlapping emission of cellulose and silica in the same wavelength range (Barud et al., 2008). The values obtained for Rh6G containing films are in good agreement with the literature (Martínez Martínez et al., 2005). The emission lifetime and quantum yield values of the composite film with higher silica content (CS4-Rh, emission monitored at 556 nm) are 4.23 ± 0.01 ns and 0.50 ± 0.05, respectively. The emission lifetime and quantum yield values obtained for the sample without silica (CNC-Rh, emission monitored at 567 nm) are 3.74 ± 0.02 ns and 0.15 ± 0.01, respectively. These results indicate that the addition of silica decreases the total amount of Rh6G dimers, since part of the dye molecules migrates to the SiO_2_ phase, leading to a dilution effect.

**TABLE 1:** Emission lifetime of CNC/silica composite films under excitation at 330 nm: the emission of cellulose (*τ_1_*) and Rh6G (*τ_2_*) was monitored at 430 and 560 nm, respectively.

Sample	τ_1_ (ns)	τ_2_ (ns)	Φ
CNC	6.83 ± 0.05	–	–
CNC-Rh	7.62 ± 0.06	3.74 ± 0.02	0.15 ± 0.01
CS1-Rh	9.47 ± 0.08	3.88 ± 0.02	0.40 ± 0.04
CS2-Rh	10.81 ± 0.09	4.21 ± 0.01	0.50 ± 0.05
CS3-Rh	12.76 ± 0.09	4.44 ± 0.01	0.65 ± 0.07
CS4-Rh	12.03 ± 0.11	4.23 ± 0.01	0.50 ± 0.05

*Absolute quantum yield values under excitation at 528 nm: emission monitored at 567 and 556 nm for CNC-Rh and Rh6G containing CNC/silica samples, respectively.*

**FIGURE 6:**
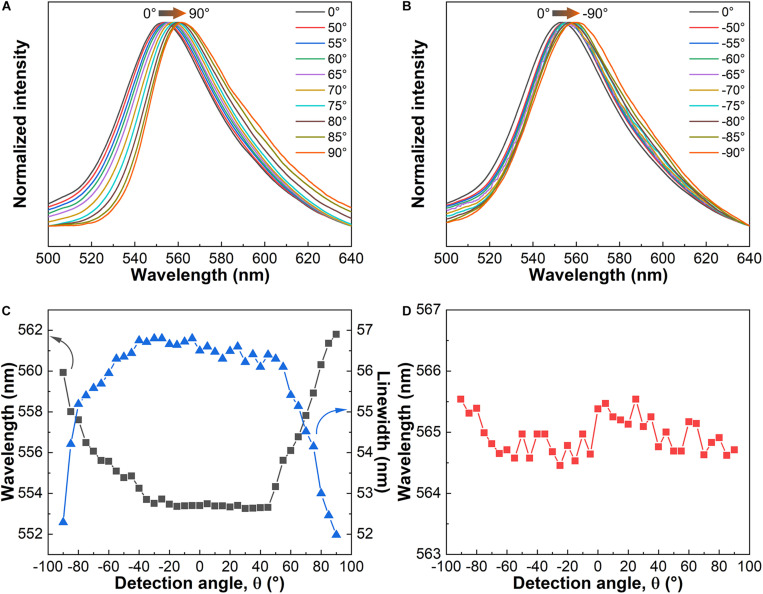
Angle-dependent emission spectra of CS4-Rh under excitation at 348 nm: −90° < θ < 0° **(A)** and 0° < θ < 90° **(B)**. Variation of the maximum wavelength (λ_0_) and linewidth of the emission bands of the CS4-Rh composite as a function of the detection angle **(C)**. Variation of λ_0_ of the emission bands of an Rh6G-containing cellulose membrane as a function of the detection angle **(D)**.

The angle-dependent emission spectra presented in Figure 6 were measured by varying the detection angle from 90° to –90°. The bandwidth of sample CS4-Rh decreases with a simultaneous redshift of λ_0_ for –90° ≤ θ ≤ –40° and 40° ≤ θ ≤ 90°. Figure 6C shows the variation of λ_0_ and the bandwidth with the detecting angle. These pronounced effects of the liquid crystal structure on the emission properties indicate potential photonic applications for these new materials. Results obtained for an Rh6G-doped achiral cellulose membrane are also shown (Figure 6D) to make clear that the photonic effects are indeed related to the unique liquid crystalline structure of CNC.

**FIGURE 7:**
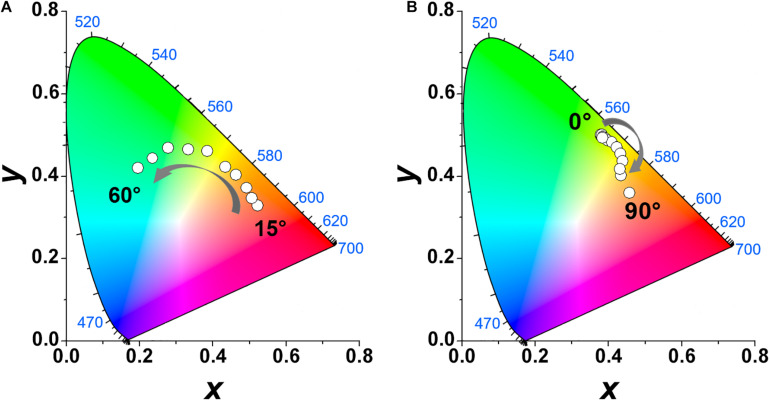
Chromaticity diagram of reflection of the CS4-Rh composite film as a function of the detection angle (0° < θ < 60^*circ*^, **A**). Chromaticity diagram of the emission of CS4-Rh composite film under excitation at 348 nm as a function of the detection angle (0° < θ < 90°, **B**).

**FIGURE 8:**
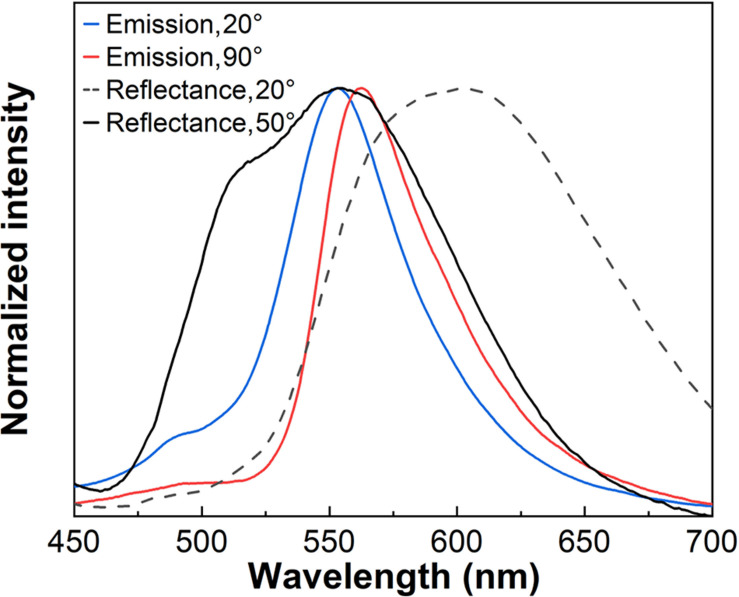
Emission spectra of CS4-Rh at 20° and 90° detection angles under excitation at 348 nm. Reflectance spectra of the same sample at 20° and 50° detection angles.

The color coordinate diagrams are seen in Figure 7. The shift observed for the emission color at different detection angles is a clear manifestation of the liquid crystal photonic properties of the host. Figure 8 shows the emission spectra obtained for the sample CS4-Rh for detection angles of 20° and 90°. Reflectance spectra are also shown for incident angles of 20° and 50°. Figure 8 intends to show that the general effect of the stopband is to blueshift the band emission compared to the one observed at 90°. It is possible to see in Figures 7, 8 that the observed emission spectra can be obtained from a convolution of Rh6G emission and the stopband due to the liquid crystal structure which changes with the detecting angle. The results obtained for the other samples are presented in Supplementary Figure S6. The same effect of the liquid crystalline structure on the emission spectra is observed. The values of *P* change by changing the silica content and the angular range where the redshift is seen in emission band changes as well. Therefore, the periodic structure with chiral nematic ordering leads to selective suppression of a range of the emitted wavelengths.

## Conclusion

Freestanding luminescent and iridescent CNC-based silica composite films were obtained from bacterial cellulose and TEOS. The tuning of the helical pitch was achieved by adjusting the relative silica content, where the incorporation of luminescent Rhodamine 6G within the periodic chiral-nematic structure allowed the modulation of the emitted light by varying the detection angle. The combination of all these features into a single material offers a novel strategy to modulate the emission of luminescent species for applications in optical devices, such as sensors, lasers, or tunable filters.

## Data Availability Statement

The raw data supporting the conclusions of this article will be made available by the authors, without undue reservation.

## Author Contributions

HB, SR, and LC conceived the initial idea. ÉP and RF designed the experimental setup. MS, FM, and LL carried out the preparation of the materials and performed the experiments. MS and FM analyzed the data and wrote the manuscript. SR, HB, LC, and RF worked on funding acquisition. All authors have read and approved the final manuscript.

## Conflict of Interest

The authors declare that the research was conducted in the absence of any commercial or financial relationships that could be construed as a potential conflict of interest.
