# Supplementary material for: Cellulose Based Photonic Materials Displaying Direction Modulated Photoluminescence
Source: Front Bioeng Biotechnol. 2021 Mar 30;9:617328. doi: 10.3389/fbioe.2021.617328 (PMC8042215; doi:10.3389/fbioe.2021.617328)
Supplement: Supplementary file 1 [file Data_Sheet_1.pdf]

Supplementary Material

1 Supplementary Figures and Tables

1.1 Supplementary Figures

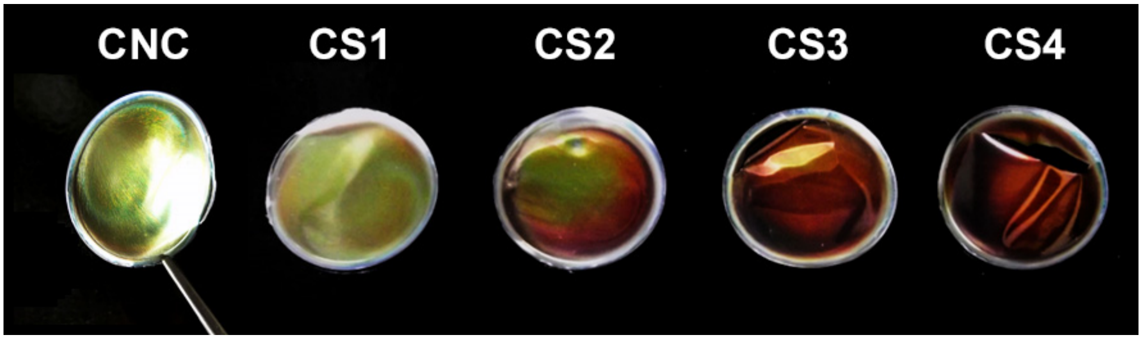

Supplementary Figure S1. Photograph of CNC/silica composite films with different contents of silica. Viewing is normal to the surface of the film.

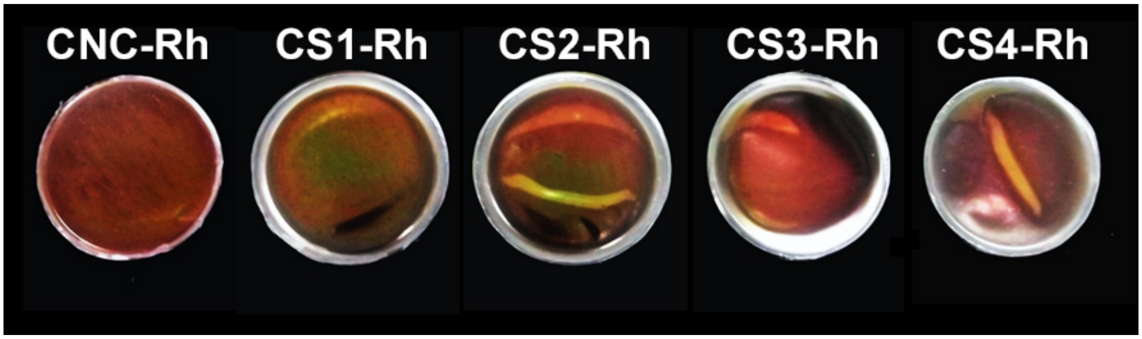

Supplementary Figure S2. Photograph of CNC/silica composite films doped with Rh6G with different contents of silica. Viewing is normal to the surface of the film.

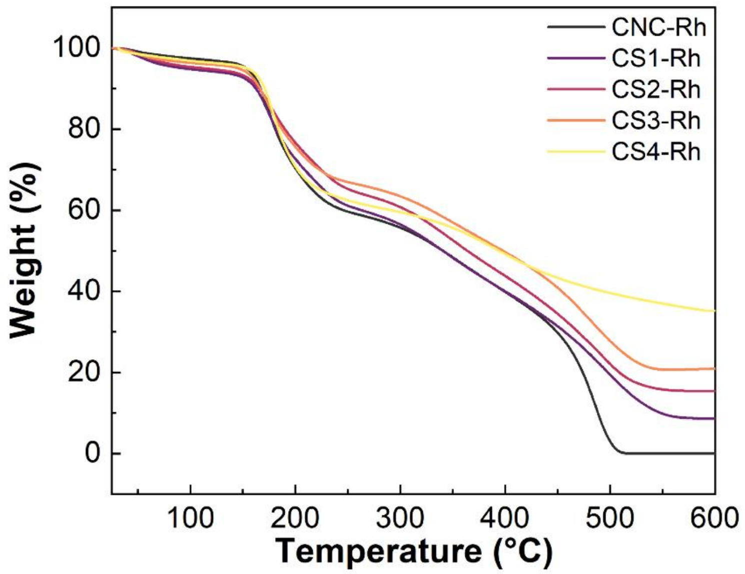

Supplementary Figure S3. TGA curves of the CNC/silica composite films doped with Rh6G.

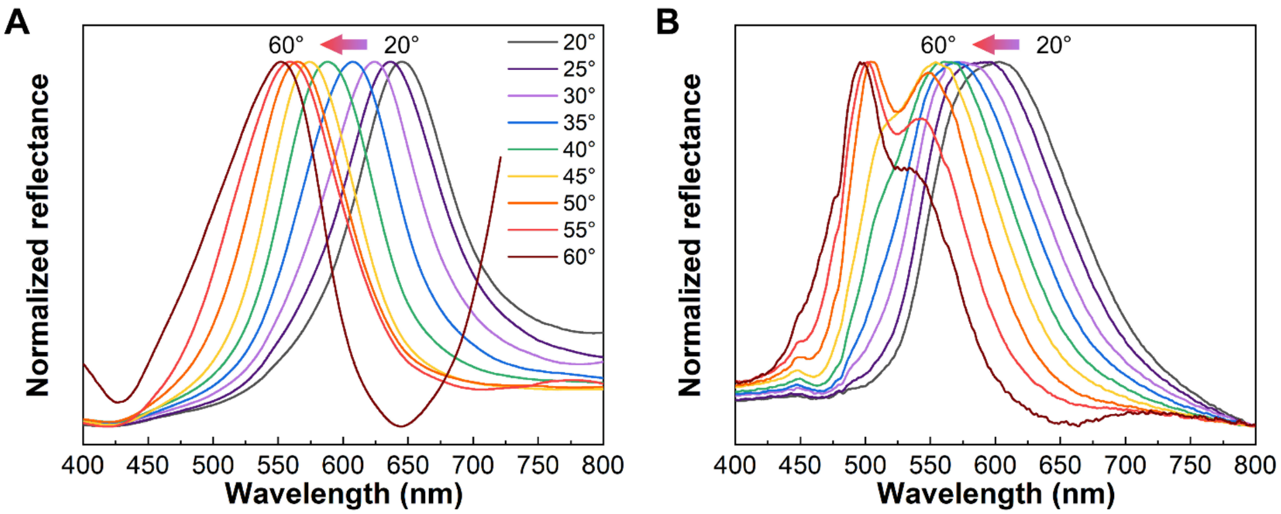

Supplementary Figure S4. Specular reflectance spectra of samples CS4 (A) and CS4-Rh (B) between 20° and 60°.

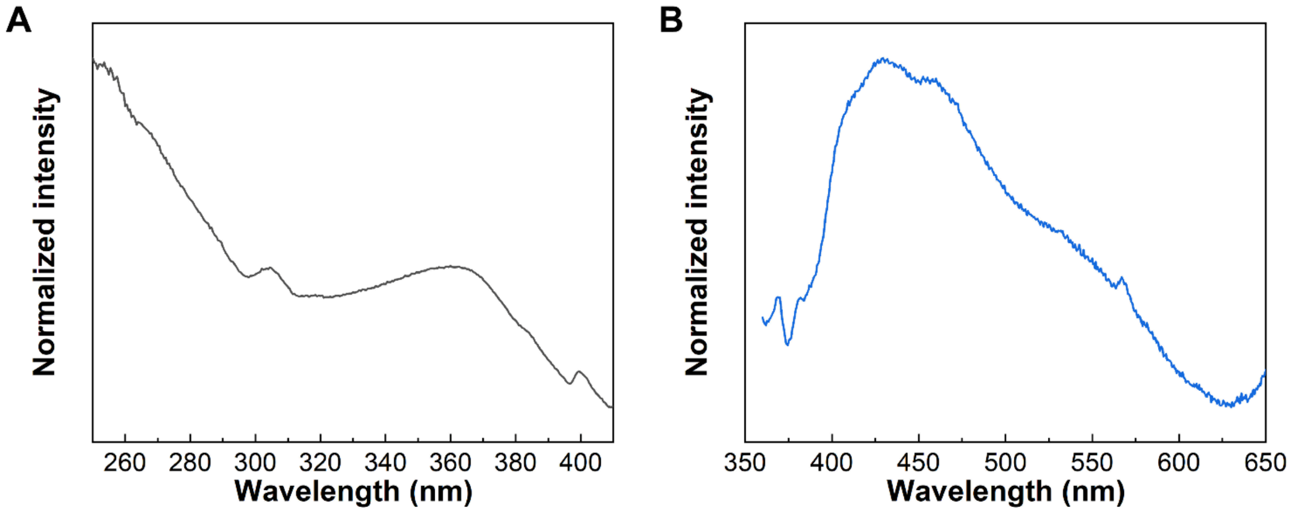

Supplementary Figure S5. Excitation spectrum monitoring emission at 425 nm (A) and emission spectrum under excitation at 345 nm (B) of pristine CNC sample.

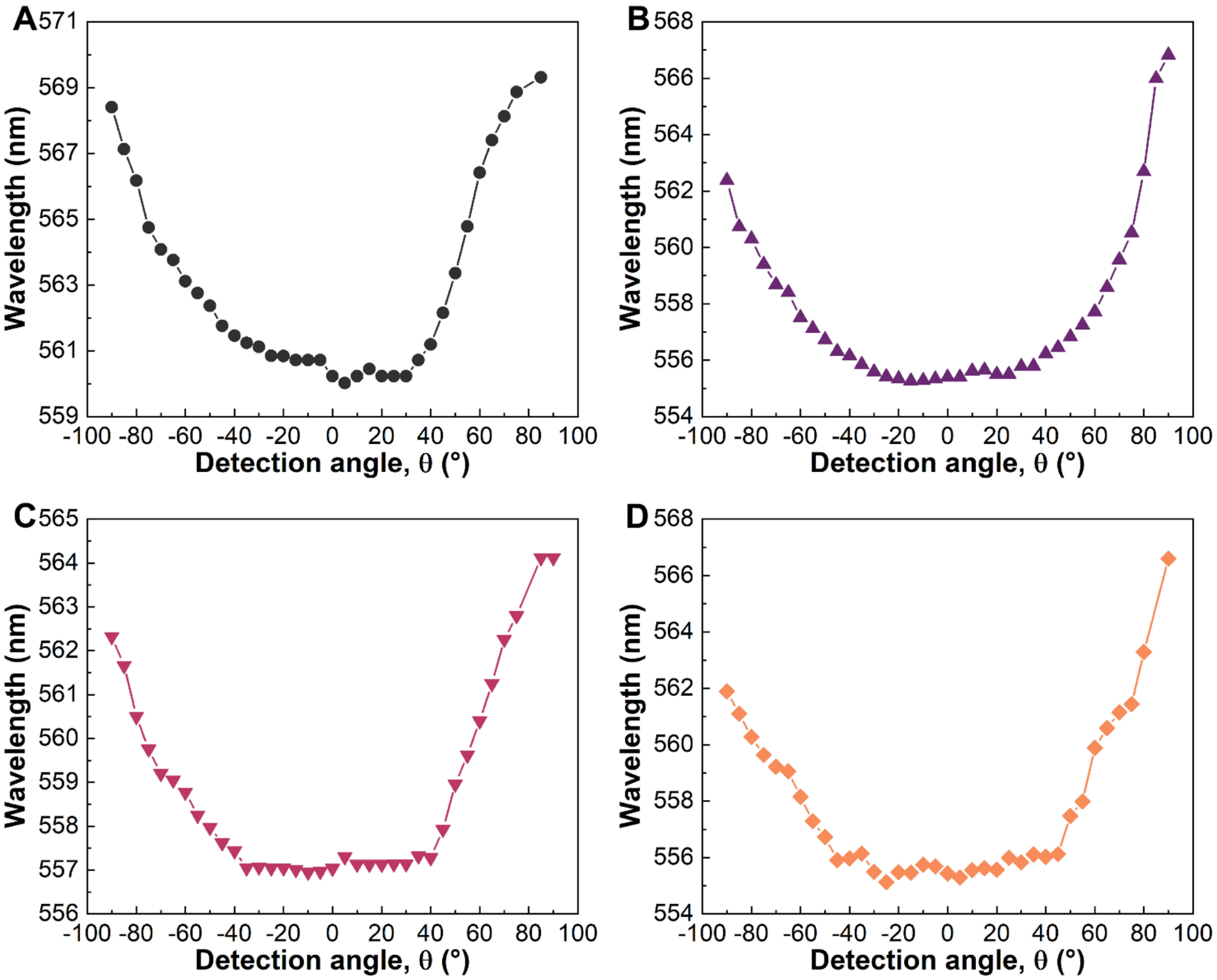

Supplementary Figure S6. Variation of the maximum emission wavelength (λ_0) of the CNC/silica composite films doped with Rh6G as a function of the detection angle (–90°< θ <90°): CNC-Rh (A), CS1-Rh (B), CS2-Rh (C), and CS3-Rh (D).

1.2 Supplementary Schemes

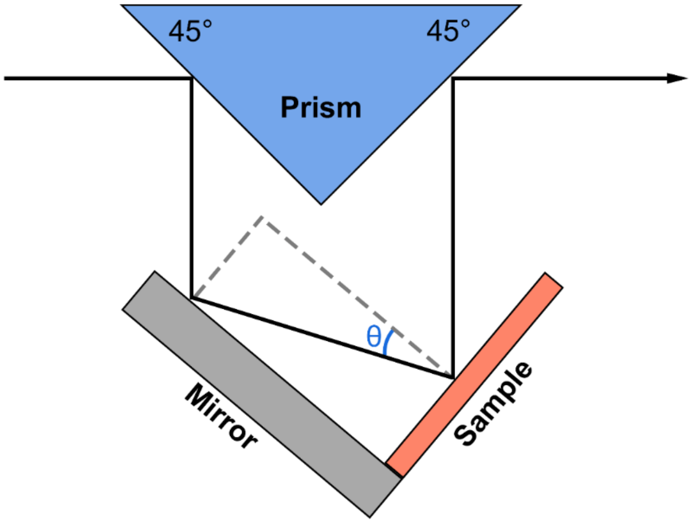

Supplementary Scheme 1. Scheme of the experimental setup of the specular reflectance measurement.

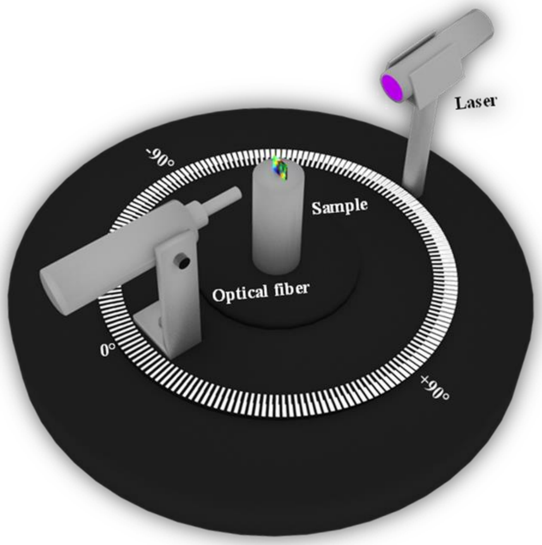

Supplementary Scheme 2. Experimental setup used to record the emission spectra as a function of the emission angle (0° < θ < –90° and –90° < θ < 0).

1.3 Supplementary Tables

Supplementary Table S1. Variation of the helicoidal pitch of the obtained films.

Samples	***P*** **– Specular reflectance (nm)**	***P*****/2 – SEM (nm)**
CNC	143 ± 4	-
CS1	122 ± 6	-
CS2	119 ± 3	-
CS3	168 ± 5	-
CS4	190 ± 8	-
CNC-Rh	141 ± 5	78 ± 1
CS1-Rh	138 ± 7	82 ± 2
CS2-Rh	140 ± 5	81 ± 5
CS3-Rh	191 ± 7	96 ± 8
CS4-Rh	240 ± 12	115 ± 3
